# Enhanced Detection of *Coccidioides* spp. Fungi from Environmental Samples Using Droplet Digital PCR

**DOI:** 10.3201/eid3204.251146

**Published:** 2026-04

**Authors:** Jessica Paulette Segovia-Mota, Ricardo Eaton-González, Jimena Carrillo-Tripp, Meritxell Riquelme

**Affiliations:** Centro de Investigación Científica y Educación Superior de Ensenada (CICESE), Ensenada, Mexico (J.P. Segovia-Mota, J. Carrillo-Tripp, M. Riquelme); Facultad de Ciencias Marinas, Universidad Autónoma de Baja California, Ensenada (R. Eaton-González)

**Keywords:** Coccidioides, Coccidioidomycosis, fungi, reemerging fungal pathogens, soil-dwelling fungi, digital droplet PCR, Mexico

## Abstract

Coccidioidomycosis (Valley fever), caused by *Coccidioides* spp. fungi, is a reemerging, neglected fungal disease endemic to arid and semiarid regions of the Americas. Environmental detection remains challenging because of spatial heterogeneity, seasonal variability, low DNA abundance, PCR inhibitors, and lack of standardized methods. We conducted environmental surveillance in Baja California, Mexico, an understudied region near the US–Mexico border, by collecting 74 soil samples from active rodent burrows across 5 locations. We evaluated droplet digital PCR (ddPCR) for *Coccidioides* detection and compared ddPCR with nested PCR targeting the internal transcribed spacer 1 region. ddPCR demonstrated greater sensitivity, detecting *Coccidioides* spp. DNA at all sampling sites, whereas nested PCR detected *Coccidioides* spp. DNA from only 1 site. Although additional work is required to rigorously quantify sensitivity and specificity, ddPCR could help identify *Coccidioides* environmental hotspots, thus enabling public health interventions, such as warning communities of areas that pose higher risk for infection.

Coccidioidomycosis, commonly known as Valley fever, has been increasingly reported across North, South, and Central America, pointing to its reemergence as a public health concern (*1*). Coccidioidomycosis is caused by 2 ascomycetous fungal species belonging to the genus *Coccidioides*: *C. immitis* and *C. posadasii* (*2*). *C. immitis* is primarily found in California, USA, whereas *C. posadasii* prevails in southern Arizona, New Mexico, and Texas, USA; parts of Central and South America (*2*–*4*); and several states in Mexico, including Sonora, Nuevo León, Coahuila, and Baja California. *Coccidioides* is considered one of North America’s most concerning endemic mycoses (*5*–*7*).

Human *Coccidioides* infection occurs primarily through inhalation of airborne arthroconidia produced during the saprophytic phase (*8*). Spores easily disperse by wind and can remain viable for long periods in arid environments (*2*). Once inhaled, arthroconidia transform into parasitic spherules that, upon maturation, release endospores, which can remain in the lungs or disseminate systemically (*8*,*9*).

Despite high incidence rates of 150,000–350,000 cases annually in the United States (*10*), environmental detection of *Coccidioides* spp. fungi remains limited. Isolation of the fungi from soil is rare because of nontargeted sampling, limited understanding of the fungus’ ecologic niche, interference from other microorganisms, and Biosafety Level 3 requirements associated with handling microbes that produce arthroconidia (*10*–*13*). Most environmental isolation attempts, including animal inoculation using soil-derived suspensions, have had low success rates (*7*,*12*).

Direct detection of *Coccidioides* spp. DNA from soil without prior fungal isolation has produced mixed results (*12*). Early studies analyzing 90 soil samples from active rodent burrows in semiarid regions of Baja California, Mexico, Valle de las Palmas (VDP) and San José de la Zorra (SJZ), used nested PCR targeting the internal transcribed spacer (ITS) 2 region (*6*). Although 32 (35.5%) samples tested positive, 13.33% were later confirmed as false-positive by BLAST analysis (*6*), reflecting high ITS2 sequence similarity among fungi and amplification of nontarget taxa. To improve specificity, Vargas-Gastélum et al. (*16*) designed primers targeting the ITS1 region. Using that approach, nested PCR identified *Coccidioides* spp. DNA in 27.5% (11/40) of soil samples, primarily from rodent burrows (*16*). Consistent with previous studies (*14*,*15*,*17*,*18*), researchers observed higher detection rates in deeper rodent burrow soils than in surface samples (*16*), likely reflecting favorable microenvironmental conditions for fungal growth and the potential role of rodents as reservoir hosts (*2*,*9*,*15*,*19*).

A previous study using ELISA further evaluated exposure to *Coccidioides* spp. in wild rodents captured from VDP (*15*). That study detected coccidioidal antibodies in Eastern deer mice (*Peromyscus maniculatus*) and desert woodrats (*Neotoma lepida*) but not in San Diego pocket mice (*Chaetodipus fallax*) or Dulzura kangaroo rats (*Dipodomys simulans*), likely because of limited cross-reactivity of secondary antibodies with Heteromyidae species.

More recently, environmental detection of *Coccidioides* spp. fungi has advanced through development of the CocciEnv quantitative PCR (qPCR)–based assay, which targets a multicopy transposable element and provides high sensitivity and specificity in soil samples (*20*–*22*). Complementary molecular approaches, such as droplet digital PCR (ddPCR), offer additional advantages, including absolute quantification and increased tolerance to PCR inhibitors, enabling detection of low-abundance targets commonly encountered in environmental matrices (*23*). ddPCR partitions each reaction into thousands of droplets, each of which acts as an independent amplification reaction (*24*). Droplets are scored as negative or positive on the basis of target amplification, generating a digital readout from which target concentration is estimated by using Poisson statistics (*25*). That approach enables absolute quantification without the need for standard curves and has been shown to improve sensitivity compared with qPCR in multiple applications. For example, ddPCR outperformed qPCR in the detection of *Aspergillus* spp. fungi in human respiratory samples (*26*) and demonstrated superior sensitivity for detecting *Tilletia controversa* fungi teliospores in soil samples, achieving detection limits ≈100-fold lower than conventional PCR and several-fold lower than real-time PCR (*27*). For *Coccidioides* spp. fungi, ddPCR has previously been used to estimate copy numbers of multicopy transposon elements from DNA extracted from *Coccidioides* spp. isolates (*28*), supporting its potential as a complementary tool to qPCR-based and nested PCR–based assays for environmental surveillance. Considering those advantages, we assessed ddPCR for detecting *Coccidioides* spp. fungi in soils from previously identified *Coccidioides*–positive sites and to screen additional potentially endemic areas of Baja California identified as hotspots for *Coccidioides* spp. fungi in recent species distribution models (*29*).

## Methods

### Sampling Sites

**Figure 1:**
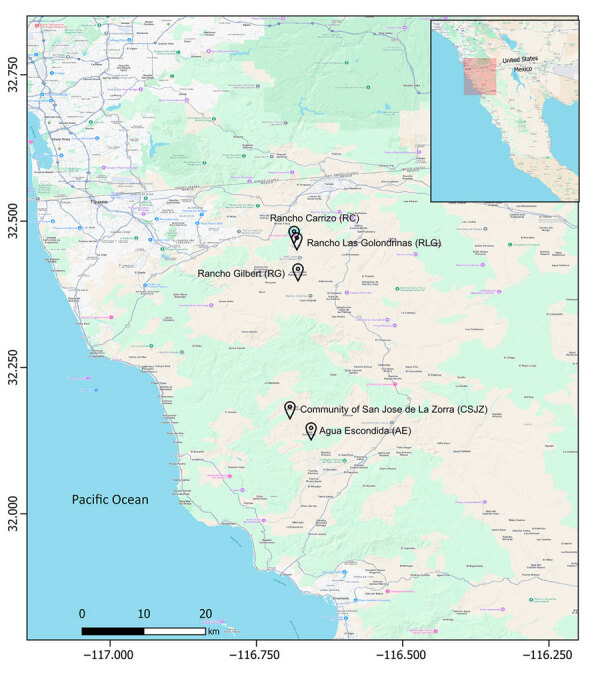
Locations of soil sampling sites from a study of enhanced detection of *Coccidioides* spp. fungi from environmental samples using droplet digital PCR, Baja California, Mexico. Rancho Gilbert was sampled on June 7, 2023; Rancho Las Golondrinas on May 16, 2024; Rancho Carrizo on May 16, 2024; and Community of San José de la Zorra and Agua Escondida on July 2, 2024. Inset shows location of study area at the US–Mexico border.

We selected 5 sampling sites within VDP and SJZ on the basis of previous reports confirming *Coccidioides* spp. detection in soil (*6*,*29*). VDP, located ≈10 km southeast of Tijuana, included 3 sampling sites: Rancho Gilbert (RG), sampled on June 7, 2023, and Rancho Las Golondrinas (RLG) and Rancho Carrizo (RC), both sampled on May 16, 2024. SJZ, situated ≈32 km north of Ensenada, comprised the Community of San José de la Zorra (CSJZ) and Agua Escondida (AE), both sampled on July 2, 2024 (Figure 1).

We collected a total of 76 soil samples: 20 from RG, 12 from RLG, 18 from RC, 14 from CSJZ, and 12 from AE (Appendix 1 Table 1). We directed sampling to active rodent burrows encountered during ≈2-hour sampling efforts at each site, guided by animal trails and visible evidence of small mammal activity. We used that strategy to maximize the likelihood of targeting ecologically relevant *Coccidioides* spp. microhabitats. We collected mixed soil samples from burrow entrances at a depth of 10–15 cm. We identified active burrows by the absence of cobwebs or vegetation obstructing the entrance and the presence of loose, uncompacted soil, indicating recent use. We collected ≈40 g of soil from each burrow and placed samples in sterile plastic jars. To prevent cross-contamination, we rinsed the sampling spoon with chlorine between sites. We used sterile gloves and face masks during sample handling as precautionary safety measures.

At each site, we recorded data using a standardized form, capturing information on global positioning system coordinates, temperature, wind speed, predominant vegetation (e.g., scrub, chaparral, forest, grassland, unvegetated or bare soil, or riparian), characteristics of the sampling site (e.g., burrow associated with or not associated with a rock or plant), soil texture (coarse, medium, fine), soil compaction (compact or uncompacted), and soil moisture condition (dry, wet, mixed). We also took panoramic site photographs and images of the specific burrow and collected sample.

### DNA Extraction from Soil Samples

We used the DNeasy PowerSoil Pro Kit (QIAGEN, https://www.qiagen.com) to extract DNA from 250 mg of soil per extraction, following the manufacturer’s protocol. We used the NanoDrop Lite spectrophotometer (Thermo Fisher Scientific, https://www.thermofisher.com) to assess DNA A260/A280 concentration and purity using 1 μL of sample. We verified DNA integrity by electrophoresis on a 1% agarose gel and normalized all DNA samples to a final concentration of 10 ng/µL.

### Detection of *Coccidioides* spp. Fungi by Nested PCR

We used nested PCR to detect *Coccidioides* from DNA extracted from soil samples. The first PCR targeted the ITS1-5.8s–ITS2 region (≈900 bp) using primers NS1 and NLB4 (*14*,*30*). We used 0.48 ng of genomic DNA from *C. posadasii* (courtesy of Dr. Bridget Barker, Institute of Pathogens and Microbiomes, Northern Arizona University, USA) as a positive control and used HPLC-grade water (highly refined Type I ultra-pure water used in high-performance liquid chromatography) as a negative control. We confirmed amplified products by electrophoresis on a 1% agarose gel. For the second round of amplification, we used a 1:10 dilution of the first PCR product. For that reaction, we used ITS1CF and ITS1CR primers to target the ITS1 region (≈120 bp) specific for *Coccidioides* spp. (*16*). We analyzed those products by electrophoresis on a 2% agarose gel. We purified positive amplicons by using a QIAquick Gel Extraction Kit (QIAGEN) and submitted amplicons to Eton Bioscience, Inc. (https://www.etonbio.com; San Diego, CA, USA), for sequencing. We manually curated sequencing results by using MEGA version 11.0.13 (https://www.megasoftware.net) and confirmed species identity via BLASTn searches against the National Center for Biotechnology Information nucleotide database (https://blast.ncbi.nlm.nih.gov).

### Detection of *Coccidioides* spp. Fungi by ddPCR

We performed ddPCR on a QX200 Droplet Digital PCR system (Bio-Rad Laboratories, https://www.bio-rad.com) after optimizing primer concentrations and annealing temperatures. We adapted the same primers used in the second nested PCR for ddPCR, following manufacturer guidelines, which recommend avoiding a G at the 5′ end, maintaining a GC content of 50%–60%, ending the 3′ end with a G or C, and avoiding primer dimer formation. Each 21-μL reaction contained 10.5 μL of QX200 ddPCR EvaGreen Supermix (Bio-Rad), 0.126 μL (60 nmol) each of forward (ITS1CF-EVA 5′-AAGTGGCGTCCGGCTGCGCACCTCCCCCGCGG-3′) and reverse (ITS1CR-EVA 5′-ACGCCGCGCAAGGCGGGCGATCCCCGGC-3′) primers, 9.248 μL of Milli-Q grade water, and 1 μL (10 ng) of template DNA. We included 2 types of negative controls: a no template control (NTC) of HPLC-grade water, and a control of 28.8 ng of *Aspergillus fischeri* DNA as a template (provided by D. Martínez-Soto, CICESE, Ensenada, Mexico). We used 0.048 ng of *C. posadasii* DNA as a template in positive controls. We generated droplets by using a QX200 Droplet Generator (Bio-Rad), transferred droplets to a 96-well plate, sealed with aluminum foil using the PX1 PCR Plate Sealer (Bio-Rad), and processed in a C1000 Touch Thermal Cycler (Bio-Rad). The amplification program consisted of an initial cycle of enzyme activation at 95°C for 10 minutes, followed by 40 cycles of denaturation at 95°C for 30 seconds, annealing/extension at 70°C for 60 seconds, and stabilization at 4°C for 5 minutes, followed by inactivation at 90°C for 5 minutes. After amplification, we read the plate by using the QX200 Droplet Reader (Bio-Rad). We performed droplet analysis by using the QuantaSoft and QuantaSoft Analysis Pro software (Bio-Rad). We manually set the threshold to differentiate positive from negative droplets on the basis of negative controls. We reported the concentration of *Coccidioides* DNA in each sample in copies/μL, calculated from the fraction of positive droplets.

One of the 3 NTCs in the ddPCR run for RG samples had a positive drop above the threshold that corresponded to 0.0663 copies/µL. Another NTC of the 3 included in the ddPCR run for samples RC, RLG, AE, and CSJZ had a positive drop above the threshold that corresponded to 0.09 copies/µL. We determined those values were background noise. For each ddPCR run, we considered samples positive only if they exhibited a concentration >2-fold higher than the maximum background value observed in the NTCs.

## Results

### DNA Yield and Purity

The average A260/A280 ratio across all samples was 1.69, and DNA purity was consistently <1.8. Although lower than ideal, those values are acceptable for downstream molecular applications like PCR and ddPCR.

### Nested PCR Results

**Figure 2:**
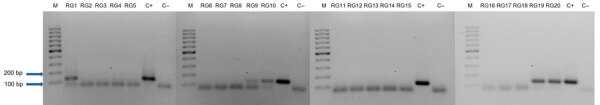
Nested PCR detection of *Coccidioides* spp. fungi DNA from environmental samples using droplet digital PCR, Baja California, Mexico. Agarose gel electrophoresis (2%) stained with ethidium bromide shows the results from nested PCR amplification of *Coccidioides* spp. internal transcribed spacer region from 20 DNA extracted from soil samples collected in RG, Baja California. Positive bands are ≈120 bp. Lanes: M, GeneRuler ladder (100-bp); RG1–RG20, DNA from soil samples; C+, positive control of 0.48 ng of *C. posadasii* DNA used as template; C–, negative control of highly refined Type I ultrapure (HPLC) water. RG, Rancho Gilbert.

Nested PCR detected *Coccidioides* spp. DNA in 5 (6.6%) of 76 soil samples, all from RG, representing 25% (5/20) of samples from that site (Table). Those positive samples (RG1, RG9, RG10, RG19, and RG20) exhibited amplicons within the expected size range of ≈120 bp (Figure 2). Sequencing and BLAST analysis confirmed all positive samples as *C. immitis.* In addition, nested PCR confirmed the positive control used throughout the experiments as *C. posadasii* (Appendix 2 Table 1).

**Table:** Comparison of nested PCR and ddPCR for detection of *Coccidioides* spp. fungi from environmental samples, Baja California, Mexico*

Location	Collection date	No. samples collected	No. (%) nested PCR–positive	No. (%) ddPCR-positive
Rancho Gilbert	2023 Jun 7	20	5 (25)	16 (80)
Rancho Carrizo	2024 May 16	18	0	5 (27.8)
Rancho Las Golondrinas	2024 May 16	12	0	1 (8.3)
San José de la Zorra	2024 Jul 2	14	0	5 (35.7)
Agua Escondida	2024 Jul 2	12	0	3 (25)
Total		76	5 (6.6)	30 (39.5)

*PCR targeted internal transcribe spacer 1 region of *Coccidioides*. ddPCR, digital droplet PCR.

### ddPCR Results

**Figure 3:**
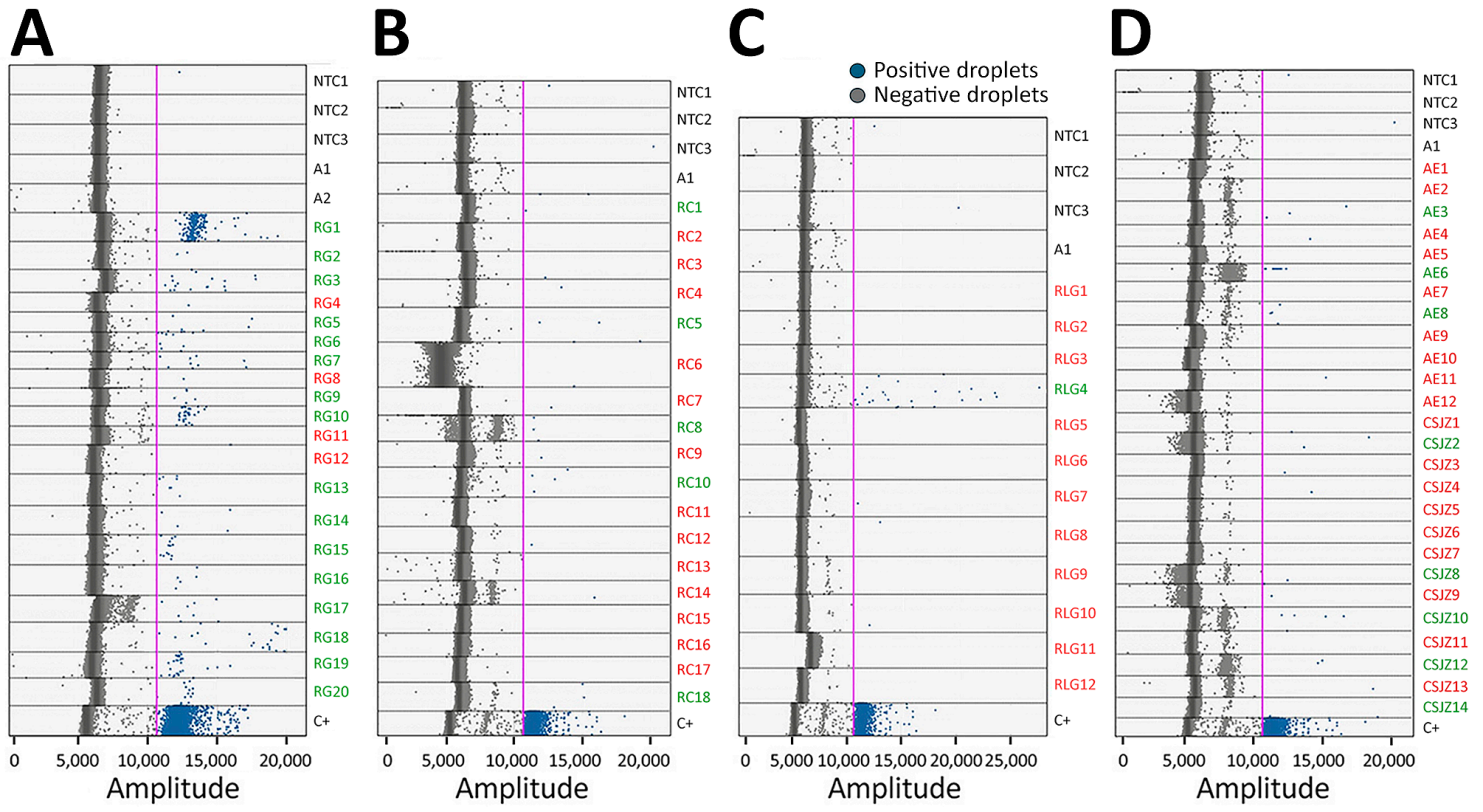
Distribution plot from droplet digital PCR analysis of *Coccidioides* spp. fungi DNA from soil samples, Baja California, Mexico. A) Samples from RG; B) samples from RC; C) samples from RLG; and D) samples from AE and Community of San José de la Zorra (CSJZ). The pink line represents the fluorescence threshold. Red text indicates negative sampling sites; green text indicates positive sampling sites. A1–2, negative templates of 28.8 ng of *Aspergillus fischeri* DNA; AE, Agua Escondida; C+, 0.048 ng of *C. posadasii* DNA used as positive template; CSJZ, Community of San José de la Zorra; NTC1–3, nontemplate controls of highly refined type I ultrapure (HPLC) water; RC, Rancho Carrizo; RG, Rancho Gilbert; RLG, Rancho las Golondrinas.

In contrast to the nested PCR, the ddPCR identified 30 (39%) of 76 soil samples as *Coccidioides* spp.–positive (Table). For all ddPCR reactions, we successfully generated >10,000 droplets per sample (Figure 3; Appendix 1 Table 2), ensuring sufficient sensitivity and robustness. Among the negative controls used for the RG samples, 2 of the 3 NTC and both *A. fischeri* DNA controls showed no positive droplets (Figure 3). However, 1 NTC (NTC1) produced a single droplet above the fluorescence threshold (Appendix 1 Table 2), likely caused by minor contamination during sample handling. Similarly, we observed isolated single-droplet signals in 2 of the 3 NTCs for the other sample sets (RC, RLG, CSJZ, and AE). On the basis of those findings, we classified samples with only 1 positive droplet as negative. Positive samples ranged from 2 droplets (2.86 copies/20-µL well) to 238 droplets (332.67 copies/20-µL well).

**Figure 4:**
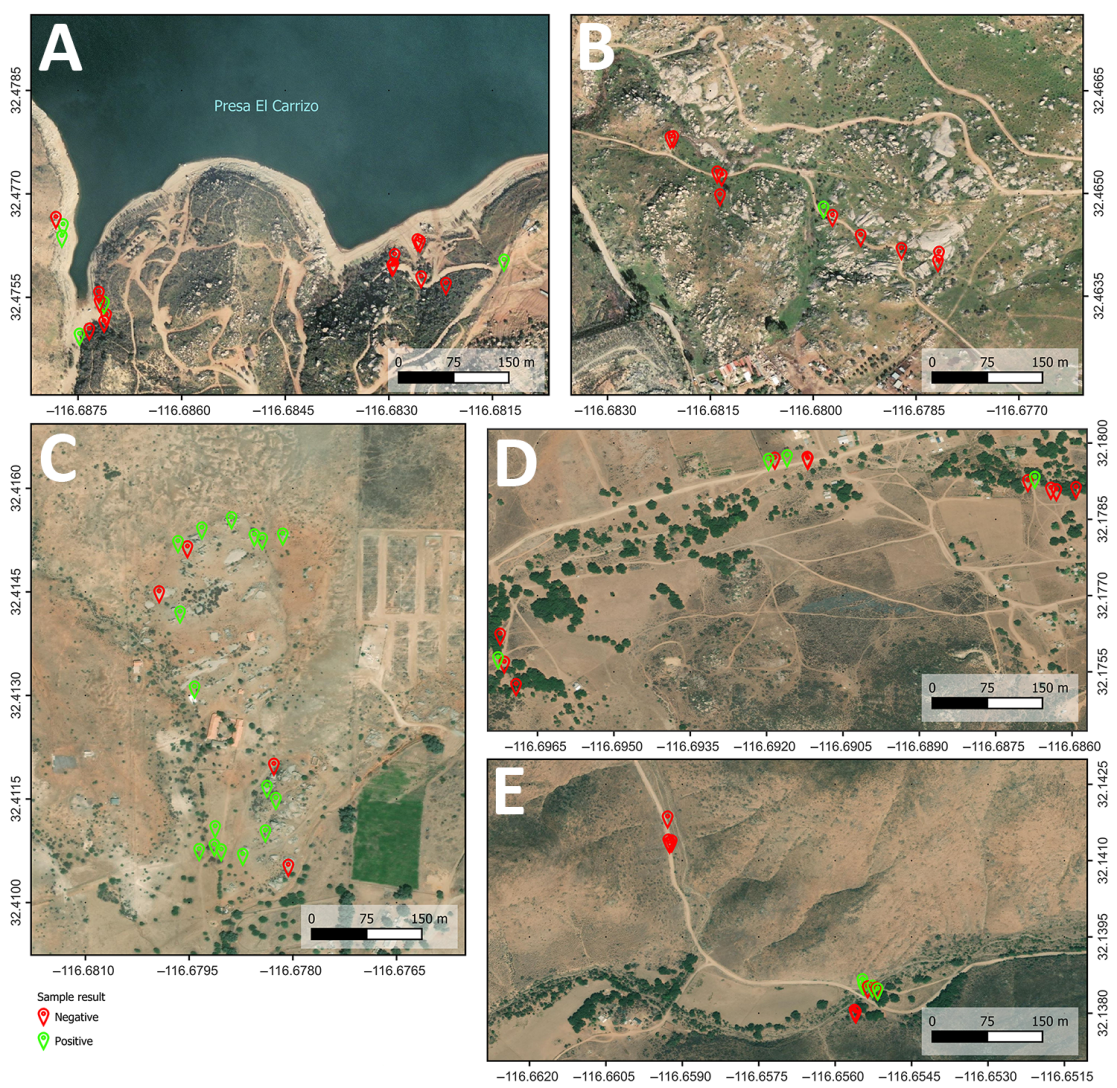
Negative and positive sites for enhanced detection of *Coccidioides* spp. fungi from environmental samples using droplet digital PCR, Baja California, Mexico. A) Rancho Carrizo; B) Rancho las Golondrinas; C) Rancho Gilbert; D) Community of San José de la Zorra; E) Agua Escondida.

Of the 20 samples from RG, 16 showed amplification with multiple positive droplets, indicating the presence of *Coccidioides* DNA (Table; Figure 3, panel A). The 5 samples identified as positive by nested PCR (RG1, RG9, RG10, RG19, and RG20) were also positive by ddPCR, confirming *Coccidioides* DNA. In addition, 11 other samples were also *Coccidioides*-positive by ddPCR, but samples RG4, RG8, RG11, and RG12 were negative by both nested PCR and ddPCR. In total, the ddPCR indicated an 80% detection rate for RG. Among the 18 samples from RC, which were all negative by nested PCR, 5 samples (RC1, RC5, RC8, RC10, and RC18) showed positive droplet amplification by ddPCR, yielding a positivity rate of 27.8% (Table; Figure 3, panel B). For RLG, only 1 (RLG4) of 12 samples tested positive by ddPCR (Figure 3, panel C), resulting in an 8.3% positivity rate. For the locality comprising CSJZ and AE, none of the 26 samples tested positive by nested PCR (Table; Figure 2). However, ddPCR detected *Coccidioides* spp. DNA in 8 of those samples (AE3, AE6, AE8, CSJZ2, CSJZ8, CSJZ10, CSJZ12, and CSJZ14), resulting in an overall positivity rate of 30.7% for that locality (Figure 3, panel D; Figure 4).

## Discussion

This study demonstrates that ddPCR is more sensitive than nested PCR for detecting *Coccidioides* spp. in environmental soil samples. ddPCR confirmed all nested PCR–positive samples from VDP and revealed additional positive samples in both VDP and SJZ, including areas previously considered *Coccidioides*-negative. Those findings highlight ddPCR’s capacity to detect cryptic environmental reservoirs of *Coccidioides* spp. fungi.

Additional support for the higher sensitivity of ddPCR comes from the amount of *Coccidioides* spp. DNA template required for successful amplification. In contrast to nested PCR, ddPCR reactions required a 10-fold dilution of *Coccidioides* spp. DNA to avoid signal saturation and droplet overloading, indicating that DNA concentrations that are readily accommodated by nested PCR exceed the optimal dynamic range of ddPCR. The necessity for dilution underscores ddPCR’s ability to detect and quantify low copy numbers of target DNA.

Our results are not intended to challenge the established CocciEnv qPCR-based method but rather to position ddPCR as a complementary molecular method for detecting *Coccidioides* spp. DNA in soil samples. Together, the 2 approaches highlight the advantage of aligning detection strategies with specific study objectives and suggest that ddPCR could complement existing qPCR-based surveillance tools rather than replace them.

Identification of *C. immitis* in the California–Baja California region is consistent with previous reports documenting the occurrence of both C. *immitis* and *C. posadasii* in northern Mexico (*4*,*16*,*31*–*33*). The coexistence of both species in that region was proposed in 2002 by Fisher et al. (*3*), when they described *C. posadasii* as distinct from the California population of *C. immitis* and documented the geographic distribution of both species across the southwestern United States and northern Mexico (*3*). Therefore, the BLAST results obtained in this study align with previous molecular and ecologic characterizations of *Coccidioides* spp. in this region.

The ability of ddPCR to detect low quantities of *Coccidioides* spp. DNA enabled us to expand the known distribution of *Coccidioides* fungi within the 2 locations studied. Nested PCR detected *Coccidioides* DNA in only 5 (6.5%) of 76 samples analyzed (all from VDP), but ddPCR confirmed those samples and detected 11 additional positive samples from VDP plus 19 additional samples from SJZ sampling locations, revealing a broader environmental range.

Previous studies using nested PCR with ITS2-targeting primers reported positivity rates of 74% in VDP and 10% in SJZ, with rodent burrow soil samples contributing to detection (*6*,*15*). Although all samples in our study were collected from active burrows, nested PCR with primers targeting ITS1 yielded lower detection rates of 25% in samples from VDP and none in samples from SJZ. Those discrepancies might reflect differences in sample size, temporal and environmental variability, or differences in primer selection and target regions (Appendix 1 Table 2). In addition, those discrepancies reinforce the need for highly sensitive detection methods, such as ddPCR, for pathogen surveillance in heterogeneous environments and for cryptic human and environmental pathogens, such as *Coccidioides* spp. fungi.

Of note, earlier studies used ITS2F and ITS2R primers (*6*,*15*), but our nested PCR protocol used the ITS1CF and ITS1CR primers developed by Vargas-Gastélum et al. (*16*). That approach enabled successful detection for *Coccidioides* spp. in 11 of 40 samples (*16*), including 9 from burrows, suggesting improved specificity for *Coccidioides* spp. using that primer set.

The ddPCR technique has previously demonstrated high sensitivity for detecting fungal pathogens such as *Pneumocystis*, *Aspergillus*, and *Cryptococcus* spp. fungi in immunocompromised patients in clinical settings (*34*). In this study, we demonstrated that ddPCR can be effectively used to directly extract DNA from soil samples to achieve sensitive and specific detection and quantification of *Coccidioides* spp. fungi in complex environmental matrices. By targeting the ITS1 region with primers optimized for ddPCR performance, our approach enabled reliable detection even when target DNA concentrations were extremely low or when PCR inhibitors (i.e., conditions that commonly limit the performance of conventional and real-time PCRs in environmental samples) were present. Those results highlight the usefulness of ddPCR as a robust tool for environmental surveillance of *Coccidioides* spp. Our findings build upon earlier work that used ddPCR to estimate the copy number of a multicopy transposon element by using DNA extracted from *Coccidioides* spp. isolates, in support of a nested qPCR (*28*). Although that study did not apply ddPCR directly to environmental DNA and encountered limitations related to reaction saturation because of the high copy number of the target (*28*), it provided a proof of concept for using ddPCR in *Coccidioides* molecular analyses. By extending ddPCR applications to soil-derived DNA and optimizing the assay for low-copy targets, our study complements and advances that prior work, demonstrating the feasibility and advantages of ddPCR for direct environmental detection and quantification of *Coccidioides* spp.

Although our ddPCR results showed clear positive results at each site, threshold determination is critical when working with environmental DNA. We manually set a fluorescence threshold to reduce false positives, excluding isolated droplets observed in negative controls. However, because of the complexity of soil matrices and the possibility of droplet misclassification or background fluorescence, sometimes referred to as the significant rainfall effect, a certain degree of false-positive results can be expected. That limitation is especially relevant because ddPCR does not permit sequencing of amplified products, which makes distinguishing true positives from potential artifacts difficult (*35*). To address that problem, we adopted a cautious interpretative approach, excluding samples with only a single droplet above the threshold. That approach minimizes overestimation, but it also raises the possibility of underreporting true positives. Nevertheless, those borderline cases could still represent genuine low-copy *Coccidioides* DNA, emphasizing the need for additional validation studies to refine ddPCR thresholds and improve specificity for environmental applications.

Most samples from AE, RC, RLG, and CSJZ showed low signal intensities, typically consisting of 2–4 positive droplets. Given the lack of technical replication, we cannot exclude the possibility that those represent false-positive samples, and we accordingly interpreted those results with caution. In contrast, sample RLG4 yielded a substantially higher signal of 26 positive droplets, providing stronger support for the presence of *Coccidioides* spp. DNA at the RLG site. That finding suggests that directed sampling with increased replication and sampling effort should be undertaken in RLG during future studies.

From a public health perspective, improved environmental *Coccidioides* detection is urgently needed amid increasing Valley fever incidence and expanding pathogen distribution, possibly linked to climate change and land use transformations (*22*,*29*,*36*). Our study represents a foundational effort to apply ddPCR for environmental detection of *Coccidioides* spp. The results highlight the method’s superior sensitivity over nested PCR and its potential for surveillance of environmental soil samples, particularly in vulnerable regions with similar ecologic characteristics, to uncover endemic areas previously undetected by conventional techniques. The enhanced sensitivity of ddPCR offers opportunities to improve spatial risk mapping, anticipate outbreak potential, and strengthen early detection strategies.

Although ddPCR entails a higher initial investment than conventional qPCR, for both instrumentation and reagents, that cost difference can be mitigated in high-throughput applications. Once equipment is in place, ddPCR becomes increasingly cost-competitive when processing large numbers of samples, particularly because it provides absolute quantification without the need for standard curves and exhibits high analytic sensitivity and run-to-run robustness. Workflows for ddPCR typically require greater technical expertise and slightly longer hands-on and processing times than qPCR. However, when assays are well optimized and performed by trained personnel, the increased sensitivity and quantitative precision of ddPCR can offset those costs by reducing the need for repeat testing and confirmatory analyses. Taken together, those features suggest that ddPCR represents a valuable and competitive option for large-scale environmental surveillance and diagnostic applications. Given its analytic robustness and sensitivity, ddPCR also has the potential to become a reference methodology in specialized clinical and environmental diagnostic laboratories, particularly for pathogens with low DNA abundance such as *Coccidioides*.

Although we created the distribution maps in this study on the basis of results from single ddPCR runs, those maps should be interpreted more generally as indicating whether a site represents a potential Valley fever hotspot, rather than as definitive or quantitative assessments of presence or absence of *Coccidioides* spp. fungi in each sampled point. As expected for environmental sampling of this organism, specific positive locations can vary across sampling times.

In conclusion, further research on ddPCR for *Coccidioides* detection using larger sample sets, additional molecular markers, and sequencing confirmation will be essential to improving detection accuracy and our understanding of *Coccidioides* spp. ecology in soil environments. Nonetheless, ddPCR could be an additional tool for detecting *Coccidioides* spp. across endemic, emerging, and reemerging regions, providing opportunities for more informed public health interventions, such as identifying high-risk environments, warning communities about areas with elevated exposure risk, and guiding occupational safety measures during soil-disturbing activities.

Appendix 1 Sampling sites from a study of enhanced detection of *Coccidioides* spp. fungi from environmental samples using droplet digital PCR.

Appendix 2 Additional information on enhanced detection of *Coccidioides* spp. fungi from environmental samples using droplet digital PCR.
