# Supplementary material for: Enhanced Detection of Coccidioides spp. Fungi from Environmental Samples Using Droplet Digital PCR
Source: Emerg Infect Dis. 2026 Apr;32(4):500–9. doi: 10.3201/eid3204.251146 (PMC13094836; doi:10.3201/eid3204.251146)
Supplement: Appendix 2 — Additional information on enhanced detection of Coccidioides spp. fungi from environmental samples using droplet digital PCR. [file 25-1146-Techapp-s2.pdf]

Article DOI: https://doi.org/10.3201/eid3204.251146

EID cannot ensure accessibility for supplementary materials supplied by authors. Readers who have difficulty accessing supplementary content should contact the authors for assistance.

Enhanced Detection of *Coccidioides* spp. Fungi from Environmental Samples Using Droplet Digital PCR

Appendix 2

Appendix 2 Table 1. Fungal species identified by sequencing of the amplicons of the ITS1 region obtained by nested PCR using as template environmental DNA from five positive soil samples from Rancho Gilbert (RG).

Sample	Fungal species of ITS1 region amplicons by soil DNA extraction	E value	% Identity	Query Cover
RG1	*Coccidioides immitis*	3e-38	98.10%	95%
RG9	*Coccidioides immitis*	1e-33	98.88%	89%
RG10	*Coccidioides immitis*	2e-36	100%	88%
RG19	*Coccidioides immitis*	2e-35	98.91%	85%
RG20	*Coccidioides immitis*	3e-31	97.59%	81%
Positive control, *C. posadasii*	*Coccidioides posadasii*	6e-40	98.06%	91%

Appendix 2 Table 2. Dates of samplings carried out by different authors.

Reference	Sampling locations	Sampling dates
Baptista-Rosas et al., 2012	Rancho Gilbert	Jun 2008
	Rancho Gilbert	Jan 2009
	Rancho Gilbert	May 2009
	Rancho Gilbert	Jun 2010
	Community of San José de la Zorra	May 2006
	Community of San José de la Zorra	Jun 2010
Catalán-Dibene et al., 2014	Rancho Gilbert	Oct 2011
